# Rare Association of Disseminated Cutaneous Leishmaniasis With Urethral Stricture: A Case Report

**DOI:** 10.7759/cureus.80179

**Published:** 2025-03-06

**Authors:** Siddharta Saxena, Vikas Kumar Panwar, Ankur Mittal, Mohammed Taher Mujahid, Mehul Agarwal, Nalin K Srivastava, Avin Singhal

**Affiliations:** 1 Department of Urology, All India Institute of Medical Sciences, Rishikesh, Rishikesh, IND

**Keywords:** disseminated cutaneous leishmaniasis, leishmania parasite infection, staged urethroplasty treatment, urethral stricture complications, urethrocutaneous fistula management

## Abstract

Leishmaniasis, a parasitic disease transmitted by sandflies, rarely presents with urogenital complications. This case highlights an unusual presentation of disseminated cutaneous leishmaniasis with urethral involvement. A 42-year-old male from an endemic region presented with macular-papules-nodular lesions on lips, nose, and tongue, accompanied by genital ulcers and urinary complications. Histopathological examination confirmed Leishmania parasites. The patient developed urethral stricture and urethrocutaneous fistula, which were managed successfully with systemic amphotericin B and staged urethroplasty. This case emphasizes the importance of considering leishmaniasis in the differential diagnosis of urogenital manifestations in endemic regions. The successful outcome demonstrates the effectiveness of combined medical and surgical management.

## Introduction

Leishmaniasis, first described by William Leishman and Charles Donovan in 1903, is a vector-borne disease affecting millions worldwide [1]. Humans contract the disease by being bitten by female phlebotomine sandflies carrying the protozoan parasite Leishmania, which is responsible for over 20 forms of the disease. An estimated 700,000 to 1 million new cases are reported worldwide each year, predominantly in tropical and subtropical regions [2].

The three primary manifestations of the disease are “visceral”, “mucocutaneous”, and “cutaneous leishmaniasis”. Cutaneous leishmaniasis (CL) is the most prevalent type and is distinguished by skin lesions that have the potential to create lasting scars [3]. Mucocutaneous leishmaniasis impacts the throat, mouth, and nose mucous membranes, whereas kala-azar, another name for visceral leishmaniasis, is the utmost critical formation, affecting internal organs [4].

Urogenital involvement in leishmaniasis is exceptionally rare. A comprehensive literature review reveals fewer than 10 documented cases of leishmanial urethritis worldwide [5]. The pathogenesis of urethral involvement likely involves direct invasion of the urethral mucosa by the parasite or extension from adjacent cutaneous lesions [5]. Previous cases reported have documented isolated instances of genital leishmaniasis, but the combination of urethral stricture and urethrocutaneous fistula represents an even rarer presentation [6].

## Case presentation

**Figure 1:**
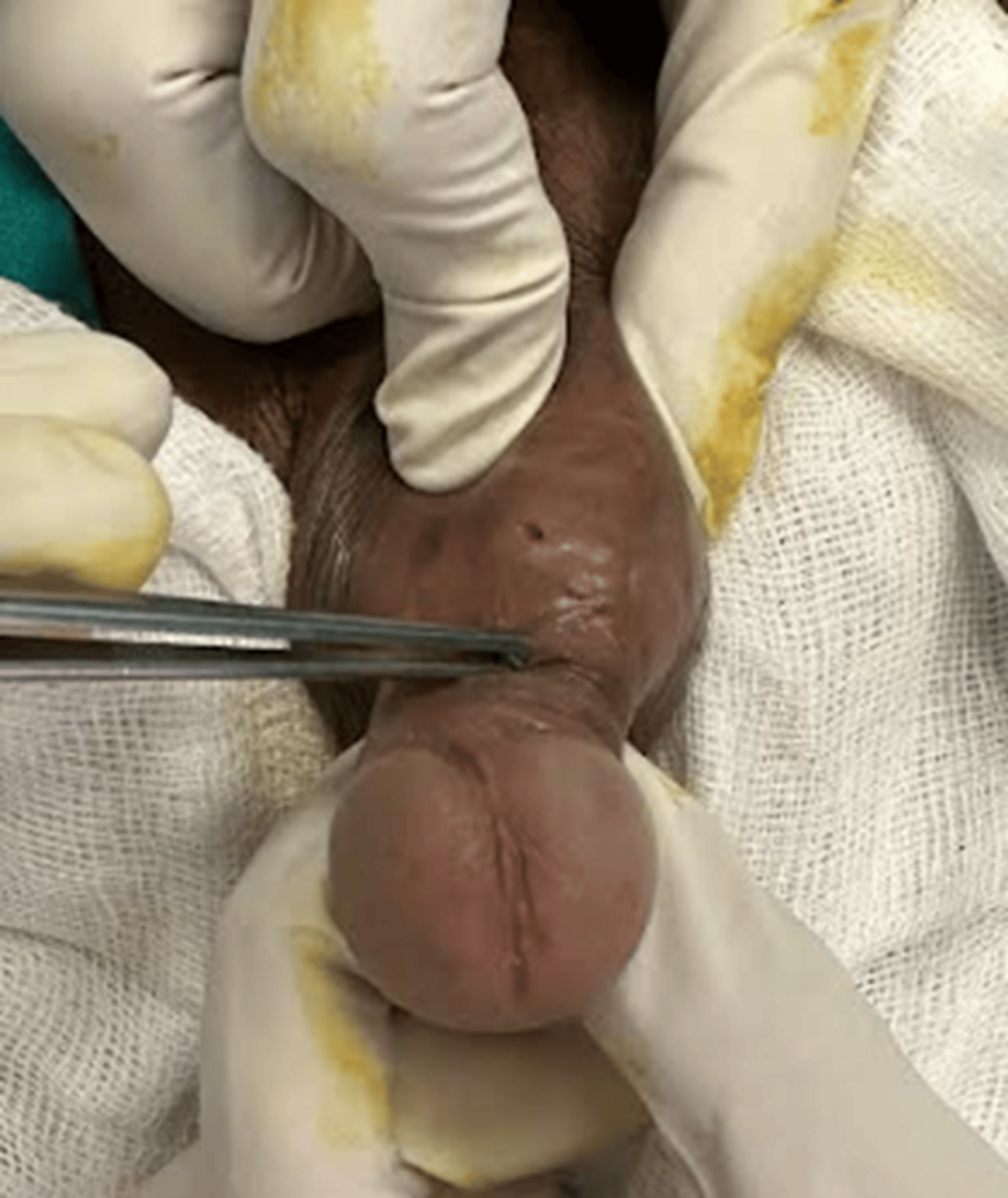
Demonstrating urethrocutaneous fistula in the patient.

A 42-year-old male, a resident of an endemic region, presented to our urology department with multiple maculopapular nodular lesions predominantly involving the lips, nose, and tongue. The patient also had painful genital ulcers, dysuria, and difficulty in urination for the past three months, which had been progressively worsening to urinary extravasation from a fistulous site on the perineum (Figure 1).

**Figure 2:**
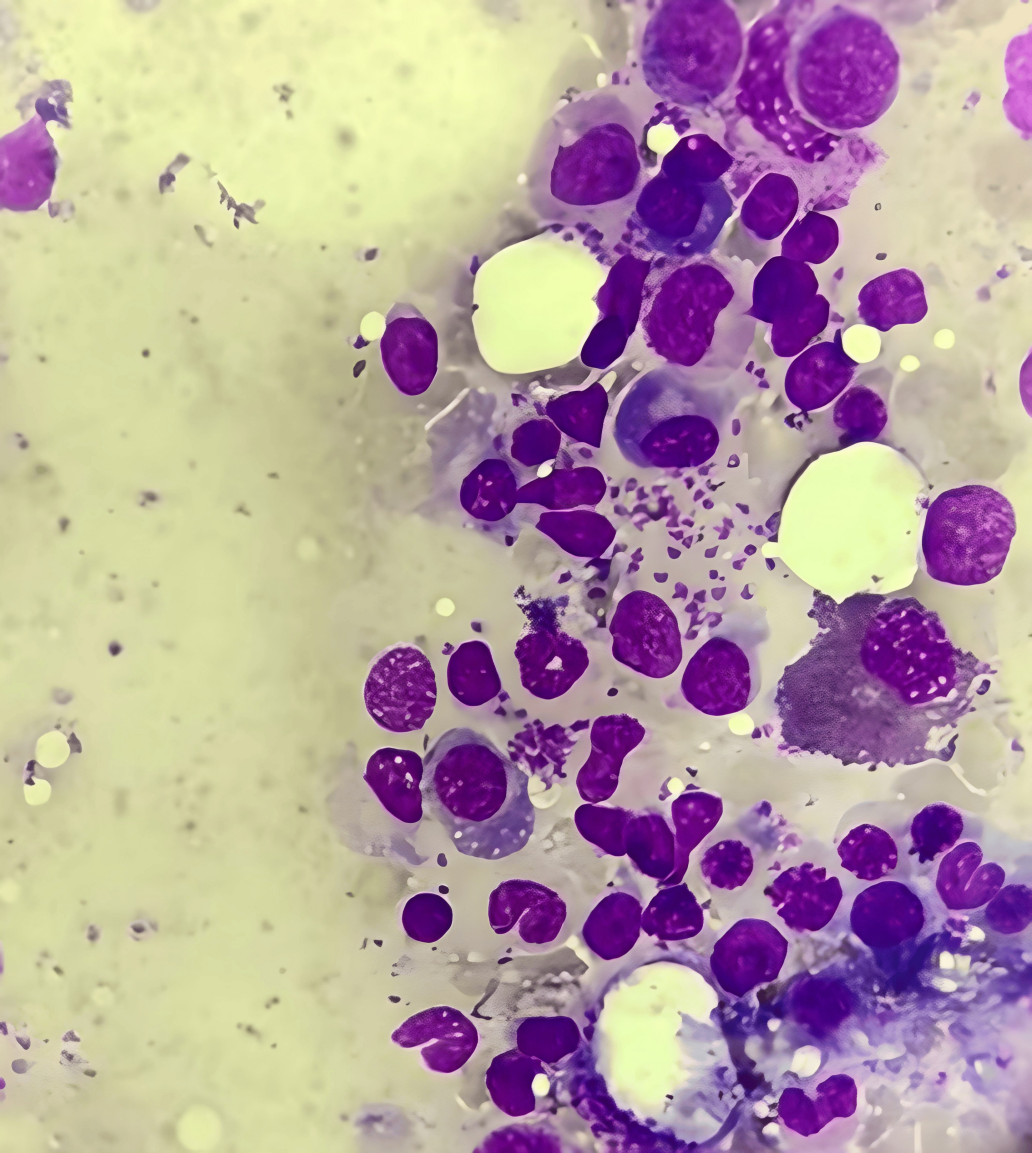
Histopathological examination showing Leishmania amastigotes within macrophages (H&E stain, 400x).

He had no history of chronic illnesses, immunosuppressive therapy, or previous genitourinary infections. However, he had a history of frequent outdoor exposure in a rural endemicity area for leishmaniasis. Physical examination revealed multiple non-healing ulcerative lesions on the external genitalia, with active urinary leakage by an abnormal opening through the perineal region consistent with urethrocutaneous fistula. A digital rectal examination was normal, and the external urethral meatus was stenotic. Routine laboratory investigations were normal with mild anemia (Hb: 10.4 g/dL), normal renal function tests, normal liver function tests, and an elevated erythrocyte sedimentation rate (ESR), suggesting an inflammatory process. Microscopic hematuria and pyuria were found on urinalysis, and the urine culture was sterile. HIV and syphilis were negative by serological tests. The cutaneous lesions were biopsied, and histopathological examination revealed Leishmania amastigotes in macrophages (Figure 2).

**Figure 3:**
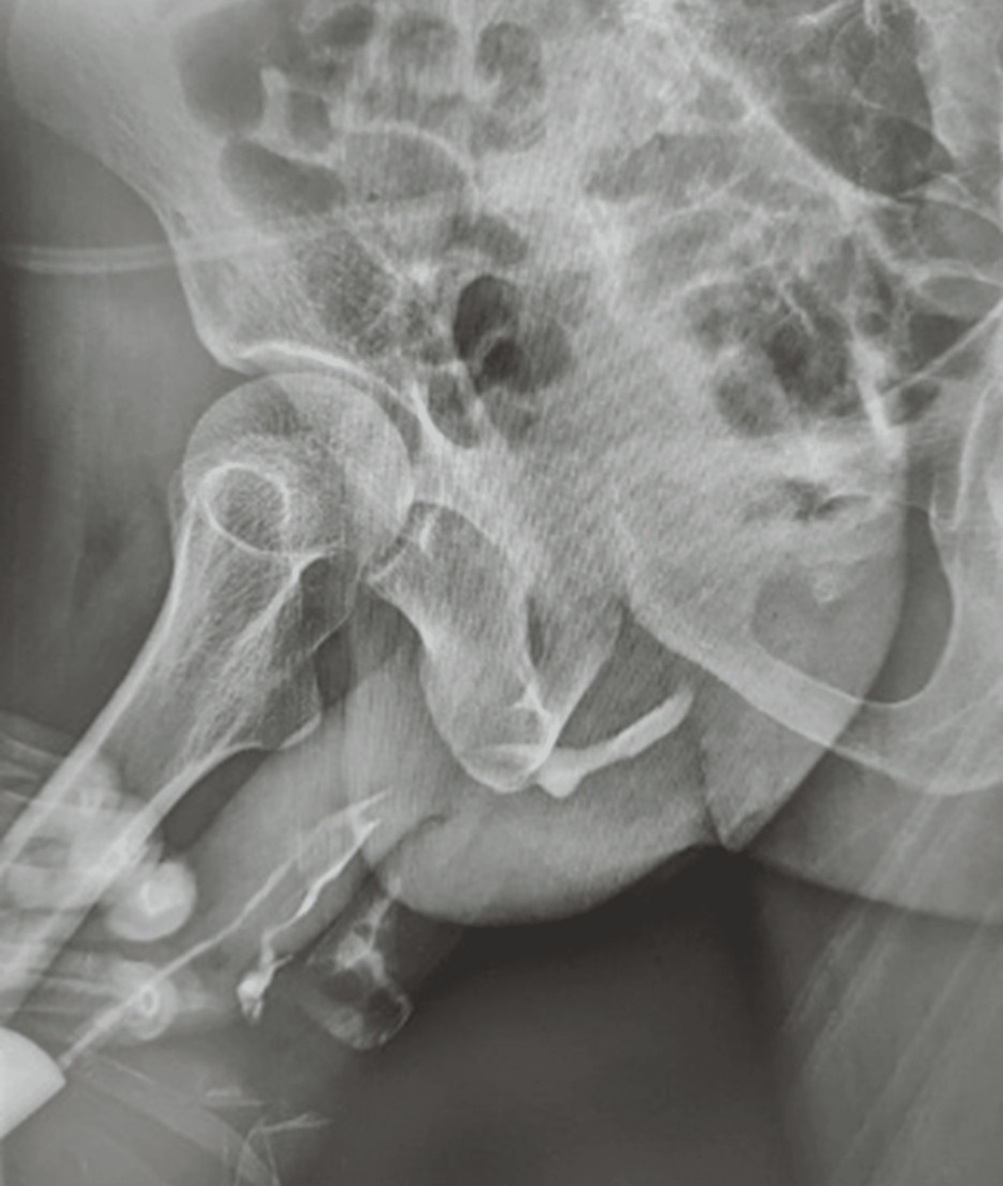
Urethral stricture was demonstrated on retrograde urethrogram with an associated fistulous tract.

Leishmania parasites were demonstrated in a slit-skin smear test. A urethrocutaneous fistula was confirmed by a retrograde urethrogram (RUG) that showed a long-segment urethral stricture with contrast extravasation at the bulbar urethra (Figure 3). 

**Figure 4:**
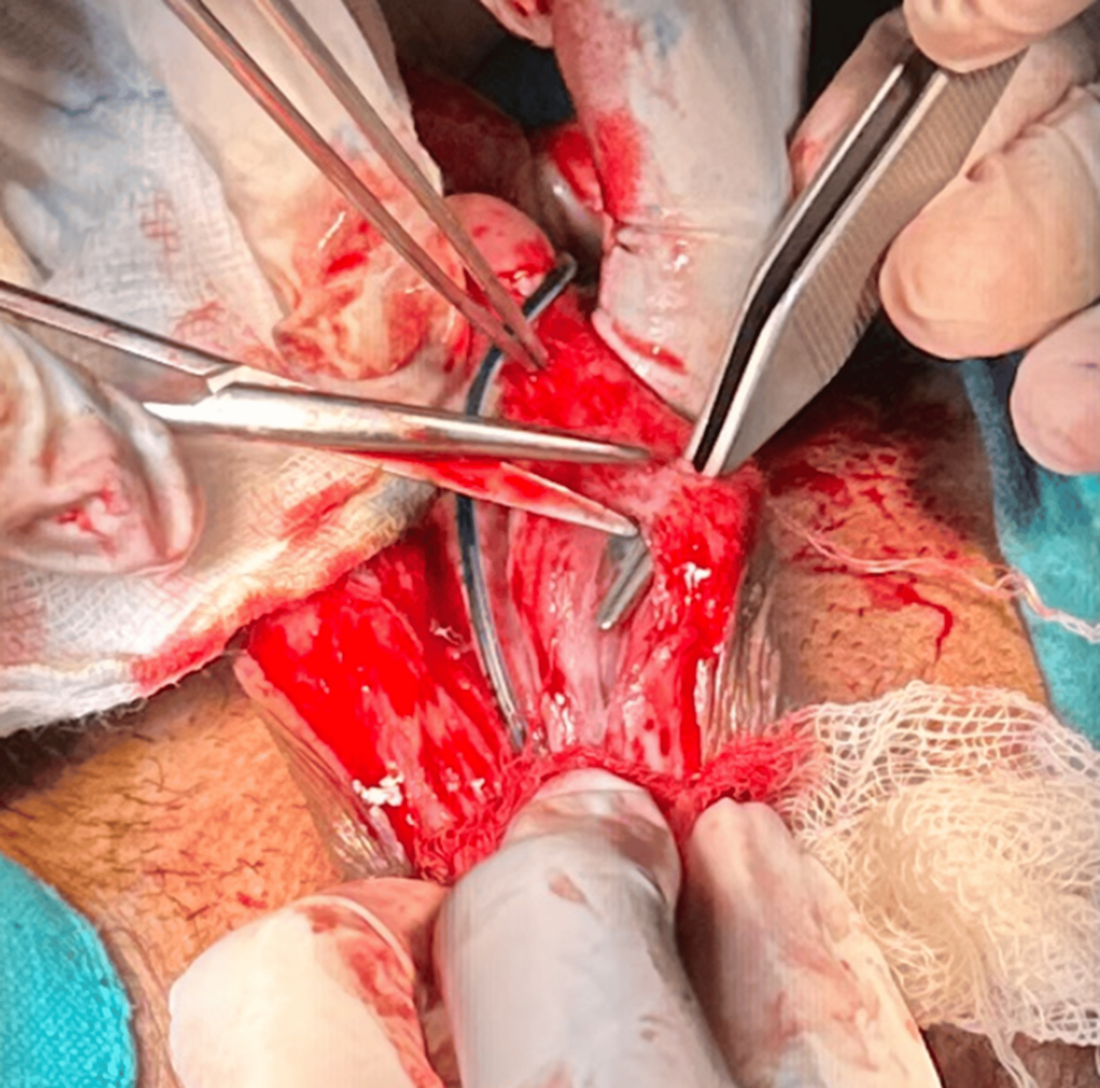
Intraoperative picture demonstrating laying open of strictured urethral segment and urethrocutaneous fistula.

Since the diagnosis of disseminated cutaneous leishmaniasis with urethral involvement was confirmed, the patient was started on systemic liposomal amphotericin B at a cumulative dose of 3 g over four weeks. Medical therapy failed to cure the persistent urinary symptoms, and so there was a need for surgery. A staged urethroplasty was planned. In the first stage, the strictured urethral segment was laid open, and the fistulous tract was carefully excised (Figure 4).

After six weeks, in the second stage, the urethra was reconstructed using a buccal mucosal graft. Post-operative recovery was satisfactory in the patient. At six months follow-up, he voided normally with no return of symptoms or urinary complications. In follow-up urethrography, a patent urethral lumen was seen, and systemic treatment resulted in complete healing of cutaneous lesions. There was no further genital ulceration or stricture recurrence.

## Discussion

This case represents a rare manifestation of leishmaniasis with urethral involvement. The literature review reveals only sporadic cases of urogenital leishmaniasis, with most reported cases originating from endemic regions. The successful management of our case adds to the limited body of evidence supporting a combined medical and surgical approach for such complications (Table 1).

**Table 1:** Summary of case presentation and management

Category	Details
Initial Presentation	• Maculo-papulo-nodular lesions on lips, nose, and tongue • Genital ulcers • Urinary complications with extravasation
Diagnostic Workup	• Biopsy confirmation of Leishmania parasites • Retrograde urethrogram showing stricture • Histopathology revealing Leishmania amastigotes
Medical Management	• Systemic amphotericin B • Total cumulative dose: 3g
Surgical Management	• Stage 1: Excision of fistulous tract • Stage 2: Buccal mucosal substitution urethroplasty
Follow-up Outcomes	• Uneventful post-operative recovery • No recurrence at six-month follow-up

The pathophysiology of urethral involvement in leishmaniasis is likely by direct parasite invasion or extension from adjacent cutaneous lesions. Early recognition and intervention are important in preventing long-term complications and attaining the best possible outcomes for these patients. In the literature, there are previous reports of leishmaniasis-related urethral involvement, although rare, that may result in significant fibrosis and persistent stricture formation. Leishmania infections are polymorphic; thus, early diagnosis is complicated, particularly in non-endemic regions, as highlighted by Georgiadou et al. (2015) [2]. Gazerani et al. (2022) also pointed out that genitourinary manifestations of leishmaniasis can mimic other chronic infectious diseases and thus delay appropriate treatment [6].

Complications require a combination of antiparasitic therapy and surgical intervention as part of the management strategies. The treatment of choice is still amphotericin B, as recommended by the WHO Expert Committee on Leishmaniasis (1990), but surgical correction is required to resolve urethral stricture [4]. Similar cases have been documented in parasitic infections by Rodrigues Coura and de Castro (2002), who have shown that staged urethroplasty can restore urethral function and minimize recurrence [3]. According to Mann et al. (2021), long-term follow-up studies are required to assess functional outcomes in patients with urogenital complications secondary to leishmaniasis [5]. A multidisciplinary approach to the diagnosis and management of such rare presentations as this case represents is also emphasized in this case to ensure timely intervention and optimize patient recovery.

## Conclusions

A multidisciplinary approach to the rare complications of leishmaniasis successfully manages this case. Early diagnosis, based on a high index of clinical suspicion, is essential in areas where atypical presentation may lead to oversight in endemic areas. Systemic antiparasitic therapy followed by staged surgical intervention has proved to be effective in rendering urinary function and preventing a poor, long-term outcome.

This case also highlights the necessity of additional investigations into the mechanisms of Leishmania persistence in genitourinary tissues and immunomodulation in treatment outcomes. Standardized management protocols for such rare presentations will require long-term follow-up and larger case series. Clinical recognition of the disease is pivotal; medico-surgical improvements have become more precise, and early diagnosis coupled with targeted medical therapy can go a distance in shaping successful patient results and improved quality of life.

## References

[1] Steverding D (2017). The history of leishmaniasis. Parasit Vectors.

[2] Georgiadou SP, Makaritsis KP, Dalekos GN (2015). Leishmaniasis revisited: Current aspects on epidemiology, diagnosis and treatment. J Transl Int Med.

[3] Rodriques Coura J, de Castro SL (2002). A critical review on Chagas disease chemotherapy. Mem Inst Oswaldo Cruz.

[4] (1990). Control of the leishmaniases. Report of a WHO expert committee. World Health Organ Tech Rep Ser.

[5] Mann S, Frasca K, Scherrer S, Henao-Martínez AF (2021). A review of leishmaniasis: current knowledge and future directions. Curr Trop Med Rep.

[6] Gazerani S, Huntington MK, Satvati J (2022). Case report and literature review: Genital leishmaniasis. IDCases.

